# The role of vasoactive intestinal peptide (VIP) in atropine-related inhibition of the progression of myopia

**DOI:** 10.1186/s12886-024-03309-9

**Published:** 2024-01-26

**Authors:** Ying Wang, Lan Li, Xiaoli Tang, Haobo Fan, Weiqi Song, Juan Xie, Yangyu Tang, Yanqing Jiang, Yunchun Zou

**Affiliations:** 1 Department of Optometry, North Sichuan Medical College, No.234 FuJiang Road, Nanchong, 637000 China; 2 Department of Ophthalmology, the Second Clinical College of North Sichuan Medical College (Nanchong Central Hospital), Nanchong, China; 3 Department of Optometry and Pediatric Ophthalmology, Ineye Hospital of Chengdu University of TCM, Chengdu, China; 4 Langzhong People’s Hospital, Langzhong, Sichuan China; 5 Department of Ophthalmology, Affiliated Hospital of North Sichuan Medical College, Nanchong, China

**Keywords:** Vasoactive intestinal polypeptide (VIP), Atropine, Form-deprivation myopia, Guinea pig

## Abstract

**Objective:**

This study aimed to investigate the potential involvement of vasoactive intestinal polypeptide (VIP) in myopia development and its contribution to the mechanism of action of the anti-myopia drug, atropine.

**Methods:**

Thirty-three-week-old guinea pigs were randomly divided into normal control (NC, *n* = 10), monocularly form-deprived (FDM, *n* = 10), and FDM treated with 1% atropine (FDM + AT, *n* = 10) groups. The diopter and axial length were measured at 0, 2, and 4 weeks. Guinea pig eyeballs were removed at week four, fixed, and stained for morphological changes. Immunohistochemistry (IHC) and in situ hybridization (ISH) were performed to evaluate VIP protein and mRNA levels.

**Results:**

The FDM group showed an apparent myopic shift compared to the control group. The results of the H&E staining were as follows: the cells of the inner/outer nuclear layers and retinal ganglion cells were disorganized; the choroidal thickness (ChT), blood vessel lumen, and area were decreased; the sclera was thinner, with disordered fibers and increased interfibrillar space. IHC and ISH revealed that VIP's mRNA and protein expressions were significantly up-regulated in the retina of the FDM group. Atropine treatment attenuated FDM-induced myopic shift and fundus changes, considerably reducing VIP's mRNA and protein expressions.

**Conclusions:**

The findings of elevated VIP mRNA and protein levels observed in the FDM group indicate the potential involvement of VIP in the pathogenesis and progression of myopia. The ability of atropine to reduce this phenomenon suggests that this may be one of the molecular mechanisms for atropine to control myopia.

## Introduction

Myopia is one of the most widely observed ophthalmic diseases, and its prevalence has increased annually in recent decades. By the year 2050, it is estimated that individuals with myopia will comprise nearly 50% of the worldwide population, with the highly myopic subgroup representing around 10% or approximately one billion individuals [1]. High myopia is typically accompanied by severe complications, including glaucoma, retinal detachment, and macular hemorrhage. As the second major cause of blindness, high myopia burdens patients, their families, and society [2, 3]. Pathogenesis of myopia is complex and relates to heredity and environment [4]. Despite numerous research efforts aimed at elucidating the underlying mechanisms of myopia, a comprehensive understanding of its pathophysiology remains elusive. This lack of knowledge significantly hinders the development of effective treatments targeting the root causes of myopia. Hence, it is imperative to persist in endeavors to comprehend the precise etiology of myopia and devise efficacious strategies for its prevention and management.

Considerable efforts have been dedicated to the prevention and mitigation of myopia. Currently, atropine is regarded as the most productive pharmaceutical agent for managing myopia [3]. This is due to its unique status as the sole treatment supported by evidence-based medicine that has demonstrated effectiveness in impeding the progression of myopia. Nevertheless, the mechanism behind myopia management continues to be a topic of debate and disagreement within the academic community. Dopamine (DA) has been shown to regulate axial elongation and slow down the development of myopia [5, 6]. But various growth factors and neurotransmitters, such as nitric oxide (NO), ZENK (Zif268, Egr-1, NGFI-A, and Krox-24), and γ-Aminobutyric acid (GABA) in the ocular tissues may also play critical roles [7–11]. Thus, multiple pathways may be involved in this complex disease.

The vasoactive intestinal polypeptide (VIP) is a 28 amino acid neuropeptide found in the brain and eyes, involved in information transmission and physiological regulation of the eyeballs and vision, among other functions. It has been shown to play an essential role in visual development and the occurrence and development of myopia [12, 13]. VIP is closely associated with the dopamine pathway. Upon connecting to its receptor, VIP exerts its physiological effects primarily through the cAMP-dependent protein kinase pathway. Additionally, VIP has modest interaction with DA in retinal cAMP level [14, 15]. Dopamine D1 receptor agonists can stimulate the secretion of VIP in the hypothalamus, while Dopamine D2 receptor agonists inhibit VIP secretion [16]. VIP can also increase the activity of tyrosine hydroxylase in the substantia nigra [17]. Tyrosine hydroxylase is responsible for catalyzing the conversion of the amino acid L-tyrosine to dihydroxyphenylalanine (dopa), which is a precursor of dopamine. However, the effect of atropine treatment on retinal VIP expression has not been reported.

We hypothesize that VIP plays an essential role in the formation of myopia, and its expression changes during the occurrence and development of myopia. Atropine's ability to control myopia may be due to direct regulation of retinal VIP levels. There are currently few publications on VIP expression in the form-deprivation myopia (FDM) model, and the research findings of different teams are not entirely uniform [18–22]. To test our hypothesis, a FDM guinea pig model was established to investigate the expression of VIP in the retina and the effects of atropine on retinal VIP levels.

## Methods

### Animal model

**Fig. 1:**
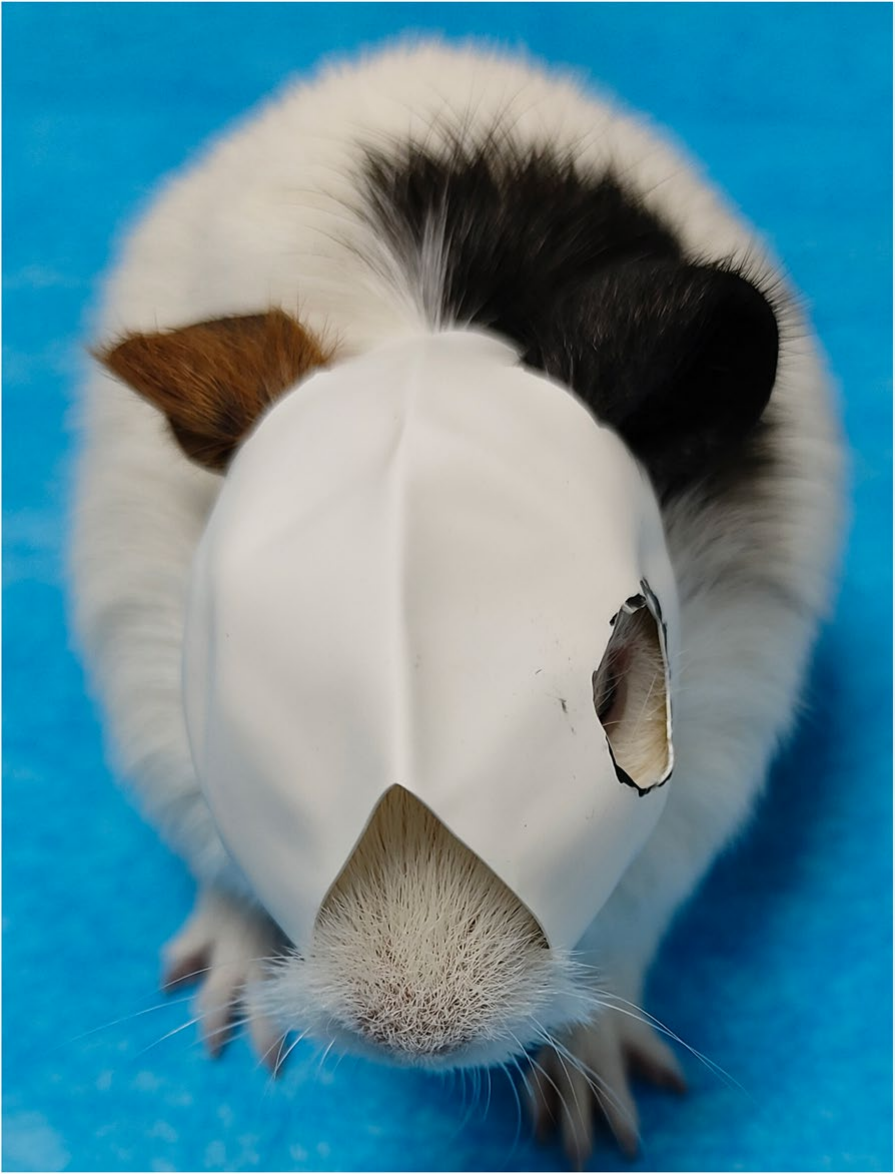
FDM guinea pig model. Cover the right eye with a semi-transparent latex balloon, while leaving the left eye, nose, mouth and ears exposed

Three-week-old pigmented guinea pigs (*n* = 30) weighing 130 ~ 150 g from the Animal Experiment Centre (North Sichuan Medical College, NanChong, China) were randomly assigned to three groups (*n* = 10 animals each): normal control group (NC, without any intervention), monocularly form-deprived group (FDM, facemasks modified from latex balloons covered the right eye [23, 24] (Fig. 1), FDM + atropine group (FDM + AT, facemasks modified from latex balloons covered the right eye and received anti-myopia treatment: treated with 1% atropine sulfate eye gel either daily). All animals were reared in the animal facilities with a 12-h light/12-h dark cycle (on at 8:00 AM and off at 8:00 PM), plenty of natural light, 50% relative humidity, and a constant temperature of 24 ± 1℃. They were fed 3 times a day to maintain sufficient food and water and received fresh vegetables and fruits three times a week as dietary enrichment. All procedures were approved by the Institutional Animal Care and Use Committee of North Sichuan Medical College (ethics review number: NSMC2022046) and adhered to the ARVO Statement for the Use of Animals in Ophthalmic and Vision Research.

### Diopter and axial length measurements

All guinea pigs were examined and excluded for eye diseases. Refractions and axial length (AL) of the right eye were measured at bi-weekly intervals after initiation of treatment (after 0, 2, and 4 weeks of form deprivation). Measurements were taken at the same time of day to avoid possible effects of circadian rhythms on eye growth. All data were measured and recorded by experienced personnel. Refractions were measured after the treatment with cycloplegia. 0.5% tropicamide eye drops (Santen, Japan) was used three times at 5-min intervals to dilate the pupil sufficiently, and then a streak retinoscope (YZ24, Suzhou 66 Vision Tech Co., Ltd., China) was performed in the dark room. The refraction data were converted to spherical equivalent (SE = spherical power + 1/2 cylindrical power). Axial length was manually measured after the treatment with 0.4% Oxybuprocaine Hydrochloride Eye Drops (Santen Pharmaceutical Co., Ltd., Japan) by A-scan ultrasonography (Quantel Medical, France). Measurements were obtained three times in a row with accuracy at 0.01 mm, and then mean values were calculated for analysis. It should be noted that the probe must be perpendicular to the central cornea, and the measurement should be gentle to avoid compression of the cornea and causing measurement errors.

### Tissue Preparation

Guinea pigs were sacrificed after anesthesia with 1% sodium pentobarbital (100 mg/kg), and then the eyeballs were rapidly enucleated on an ice plate. Every eyeball was fixed for 48 h at 4 °C in a special eyeball fixator solution (Phygene Life Sciences Co., Ltd., China, PH1848), and then dehydrated, cleared in absolute alcohol and xylene and finally paraffin-embedded. The tissues were sectioned along the long longitudinal axis with a thickness of 5 μm. The sections were stained with hematoxylin and eosin (H&E) staining to observe the histopathological changes of the retina, choroid, and sclera, and the contents of the VIP retina were detected by immunohistochemistry and in situ hybridization.

### Hematoxylin–eosin staining (H&E)

HE staining kit was used (Beijing Solarbio Science &Technology Co., Ltd., China, G1120). The slices were dewaxed into water, stained for 1 min hematoxylin staining solution, rinsed with tap water for 5 min, then differentiated in 1% hydrochloric acid ethanol for 3 s, and rinsed with tap water for 5 min. Eosin solution was added to the dye for 1 min and rinsed for 5 min. Then the slices were dehydrated in graded ethanols to 100% and cleared with xylene, sealed with neutral gum (Beijing Solarbio Science &Technology Co., Ltd., China, G8590) and a microphotographic system (Leica DM 2500, Germany) was used to observe the histopathological changes.

### Immunohistochemistry(IHC)

The slices were dewaxed into water, blocked with 5% Albumin from bovine serum (BSA) blocking solution, and 3% hydrogen peroxide solution was added to eliminate the endogenous peroxidase activity. Then the primary antibody (An anti-rat VIP antibody)(Boster Biological Technology Co., Ltd., China, PB1017) was added and incubated overnight at 4℃, and the second antibody (Goat anti-rabbit & mouse Horseradish Peroxidase (HRP) labeled polymer) was added and incubated at room temperature for 30 min. The negative control used phosphate-buffered saline instead of the primary antibody. After Diaminobenzidine (DAB) (Proteintech Group, Inc., China, PK10006) and hematoxylin (Beijing Solarbio Science &Technology Co., Ltd., China, G1120) staining, gradient ethanol dehydration, and xylene clearance, they were sealed with neutral glue. Image acquisition and analysis were performed with Image-Pro Plus.

### in situ hybridization(ISH)

The slices were dewaxed into water, and the endogenous peroxidases were eliminated by adding a 3% hydrogen peroxide solution. The nucleic acid fragments of mRNA were exposed by proteinase K (20 μg/ml) diluted with 3% citric acid. After prehybridization, the hybridization solution containing the VIP mRNA probe (5`-AGTGA GGTTG GATGA CAGGC TGCAG TTCGA AGGAG CAGGT-3`; 5`-ATTAT GATGT GTCCA GAAAT GCCAG GCATG CTGAT GGAGT-3`; 5`-GACAC TCTGA TGCAG TCTTC ACAGA TAACT ACACC CGCCT-3`) was added and and incubated overnight at 37℃. Then, BSA blocking solution was added, followed by mouse anti-digoxigenin-labeled peroxidase (Boster Biological Technology Co., Ltd., China, MK3902-R). After DAB (Proteintech Group, Inc., China, PK10006) and hematoxylin (Beijing Solarbio Science &Technology Co., Ltd., China, G1120) staining, gradient ethanol dehydration, and xylene clearance, they were sealed with neutral glue. Image acquisition and analysis were performed with Image-Pro Plus.

### Measure the mean optical density

DM-2500 (Leica) as an imaging system and DM-750 (Leica) as a microscope were used to collect images. Before the integrating optical density (IOD) measurement, the collected images were calibrated by Image-Pro plus 6.0 for optical density. Three field-of-view areas were randomly selected in each image for measurement, and the IOD of these areas was calculated. Then, the mean optical density = IOD /area was calculated.

### Statistical analysis

SPSS 23.0 statistical software was used for statistical analysis. Data were recorded with mean ± standard deviation ( X ± s). One-way ANOVA with the Least significant difference post-hoc test (LSD post-hoc test) was used to compare the differences between each group and changes over time in interocular differences across the three groups for various ocular parameters. Independent t-test was used to compare the right eye parameters between any two groups. For all data analyses, *p*-value < 0.05 was defined as significant, and *p*-value < 0.01 was considered highly significant.

## Results

### Effect of atropine on refractive power and axial length

**Fig. 2:**
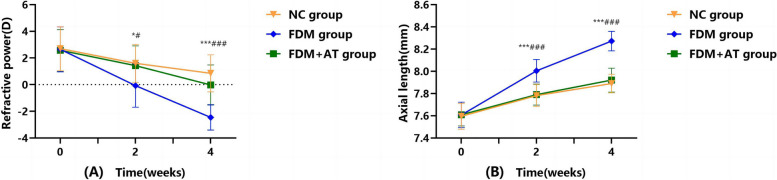
Changes in the refractive power (**A**) and axial length (**B**) in the right eyes of the different groups (mean ± SD). Compared with the control group at the same time point,*P < 0.05, **P < 0.01, ***P < 0.001. Compared with the FDM group at the same time point,^#^*P* < 0.05, ^##^*P* < 0.01, ^###^*P* < 0.001

In general, there are no significant differences in the baseline refractive error (F = 0.015, *P* > 0.05) and axial length (F = 0.054, *P* > 0.05) among the different treatment groups. Still, both myopic degree and axial length parameters increased significantly with the age of the guinea pigs for all groups. Four weeks after the onset of the experiment, the refractive error of the control (NC) group changed from hyperopia to emmetropia, and the axial length increased with age. In contrast, the FDM group displayed a substantial shift in myopia, as evidenced by significant increases in both the degree of myopia and axial length (all, *P* < 0.001). Atropine treatment of FDM guinea pigs (FDM + AT) reduced the myopic degree and axial length (all, *P* < 0.01), such that there was no significant difference between the FDM + AT and NC groups (all, *P* > 0.05). The findings of this study suggest that the administration of atropine has the potential to mitigate the alterations in refractive error and axial length produced by FDM (Table 1 and Fig. 2).

**Table 1:** Changes in the refractive power and axial length in the right eyes of the different groups (mean ± SD)

	**Refractive power(D, *n* = 10)**					**Axial length(mm, *n* = 10)**				
**Time**	**NC**	**FDM**	**FDM + AT**	**F**	**P**	**NC**	**FDM**	**FDM + AT**	**F**	**P**
0 weeks	2.700 ± 1.657	2.65 ± 1.688	2.575 ± 1.555	0.015	0.985	7.595 ± 0.116	7.608 ± 0.114	7.610 ± 0.101	0.054	0.947
2 weeks	1.600 ± 1.425^#^	-0.075 ± 1.616*	1.425 ± 1.486^#^	3.712	0.038	7.782 ± 0.099^###^	8.005 ± 0.102***	7.791 ± 0.095^###^	16.415	0.000
4 weeks	0.850 ± 1.385^###^	-2.450 ± 0.956***	-0.025 ± 1.502^###^	17.228	0.000	7.890 ± 0.084^###^	8.272 ± 0.087***	7.921 ± 0.107^###^	51.664	0.000
F	3.878	30.645	7.399			22.079	107.888	23.834		
P	0.033	0.000	0.003			0.000	0.000	0.000		

Compared with the NC group at the same time point,*P < 0.05, **P < 0.01, ***P < 0.001. Compared with the FDM group at the same time point,^#^*P* < 0.05, ^##^*P* < 0.01, ^###^*P* < 0.001

### Effect of atropine on the morphology of the eye

**Fig. 3:**
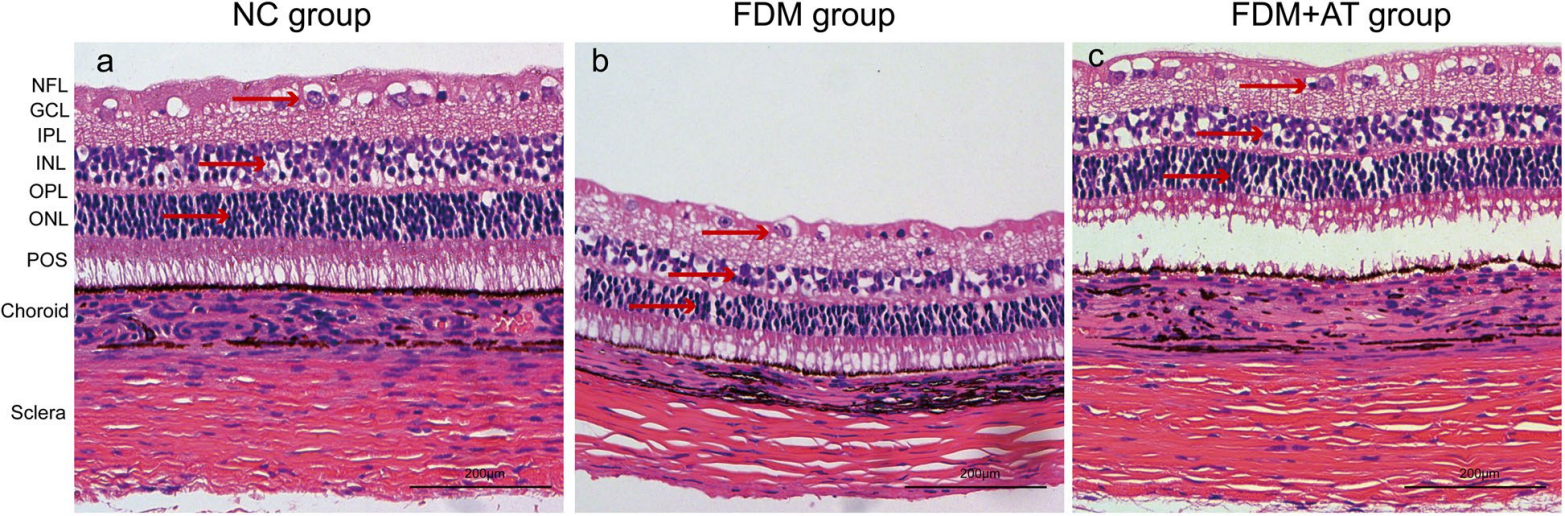
Observe the morphological structure of the Guinea pig retina, choroid, and sclera under the light microscope (HE × 200). **a** The NC group. **b** The FDM group. **c** The FDM + atropine group. NFL: nerve fibers layer; GCL: ganglion cell layer; IPL: inner plexiform layer; INL: inner nuclear layer; OPL: outer plexiform layer; ONL: outer nuclear layer; POS: outer photoreceptor segment

**Fig. 4:**
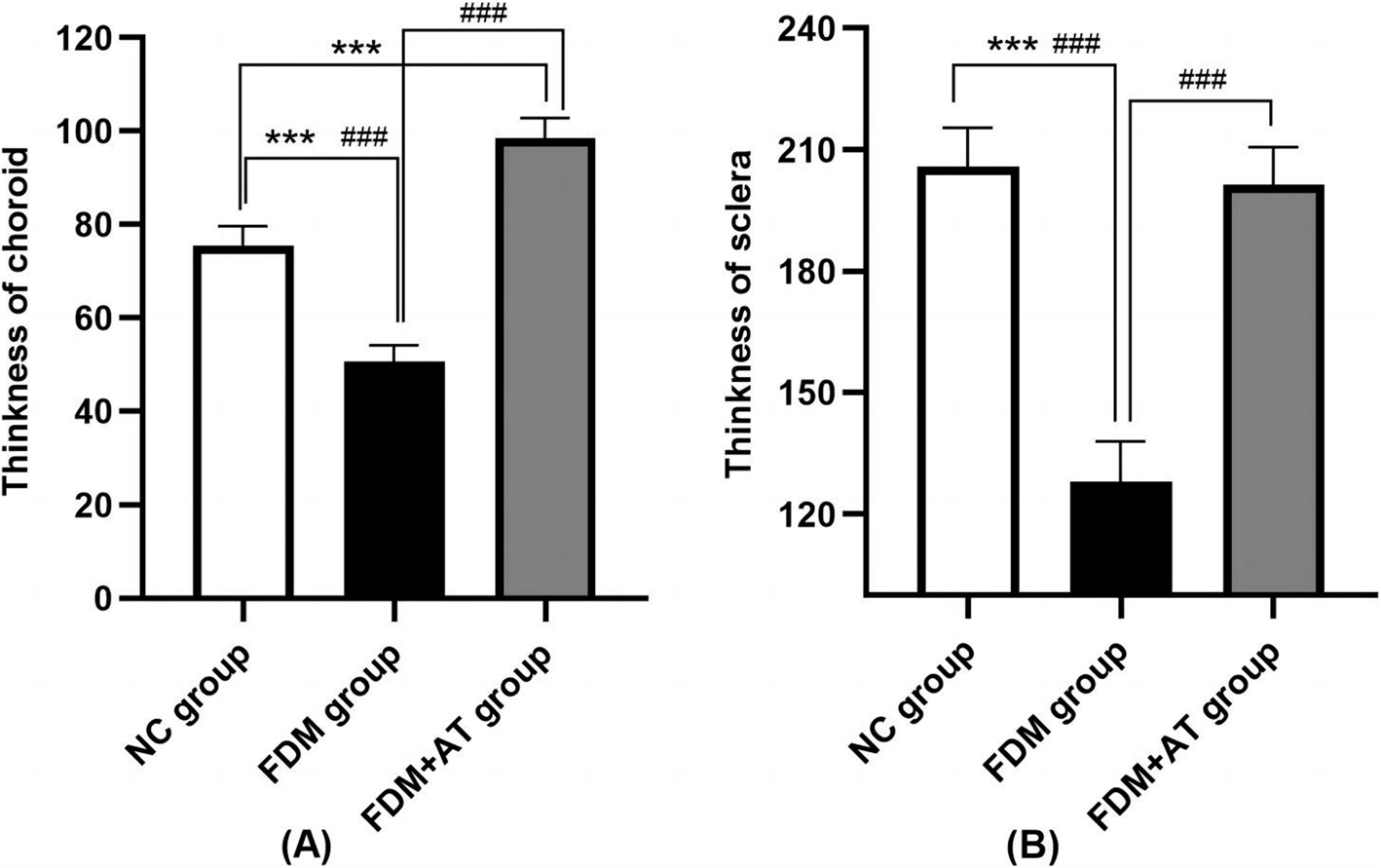
The thickness of choroid (**A**) and sclera (**B**) in different groups of guinea pigs (mean ± SD). Compared with the control group at the same time point,*P < 0.05, **P < 0.01, ***P < 0.001. Compared with the FDM group at the same time point,#*P* < 0.05, ##*P* < 0.01, ###*P* < 0.001

Four weeks after the initial phase of the study, the morphological characteristics of the retina, choroid, and sclera of all treatment groups were examined under a light microscope. The group from NC demonstrated distinct retinal layers, characterized by a well-organized and densely packed arrangement of retinal ganglion cells, cells of the inner and outer nuclear layers, and the scleral fibers. Compared with the control, the FDM group showed apparent changes in the retina, choroid, and sclera: the retinal ganglion cells and the cells of the inner and outer nuclear layers are disordered; the choroidal thickness (ChT), blood vessel lumen, and area are decreased; the sclera is thinner, with disordered fibers and increased interfibrillar space. Atropine treatment reduced damage to the retina and sclera such that the FDM + AT group is not significantly different compared with the control. In contrast, the choroidal thickness, blood vessel lumen, and area of the FDM + AT group increased significantly compared with other groups (Table 2, Figs. 3 and 4).

**Table 2:** Thickness of choroid and sclera of guinea pig

	NC	FDM	FDM + AT
Choroidal thickness	75.584831 ± 4.061680^###^	50.614680 ± 3.497180^***^	98.436654 ± 4.388368^***###^
Scleral thickness	205.891324 ± 9.525299^###^	127.918865 ± 9.967032^***^	201.452932 ± 9.200460^###^

Compared with the NC group,*P < 0.05, **P < 0.01, ***P < 0.001. Compared with the FDM group,^#^*P* < 0.05, ^##^*P* < 0.01, ^###^*P* < 0.001

### Effect of atropine on the mRNA and protein expressions of retinal VIP

**Fig. 5:**
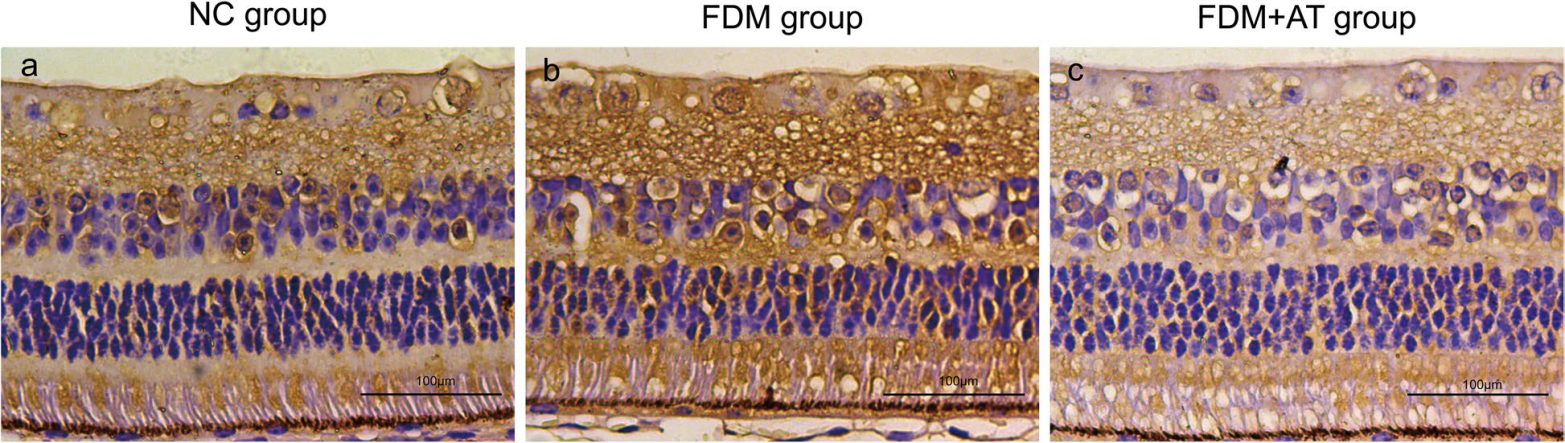
Immunohistochemical performance of retinal VIP protein in guinea pigs (DAB, X400). **a** The NC group. **b** The FDM group. **c** The FDM + atropine group. The VIP protein positive expression was brown and yellow

**Fig. 6:**
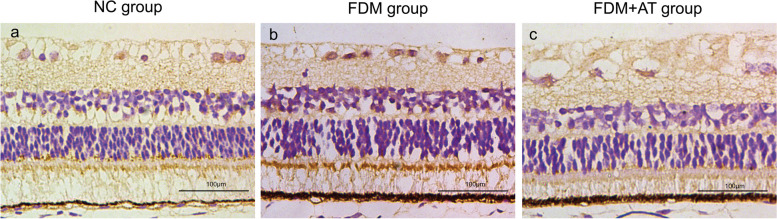
In situ hybridization performance of retinal VIP mRNA in guinea pigs (DAB, X400). **a** The NC group. **b** The FDM group. **c** The FDM + atropine group. The VIP mRNA positive expression was brown and yellow

**Fig. 7:**
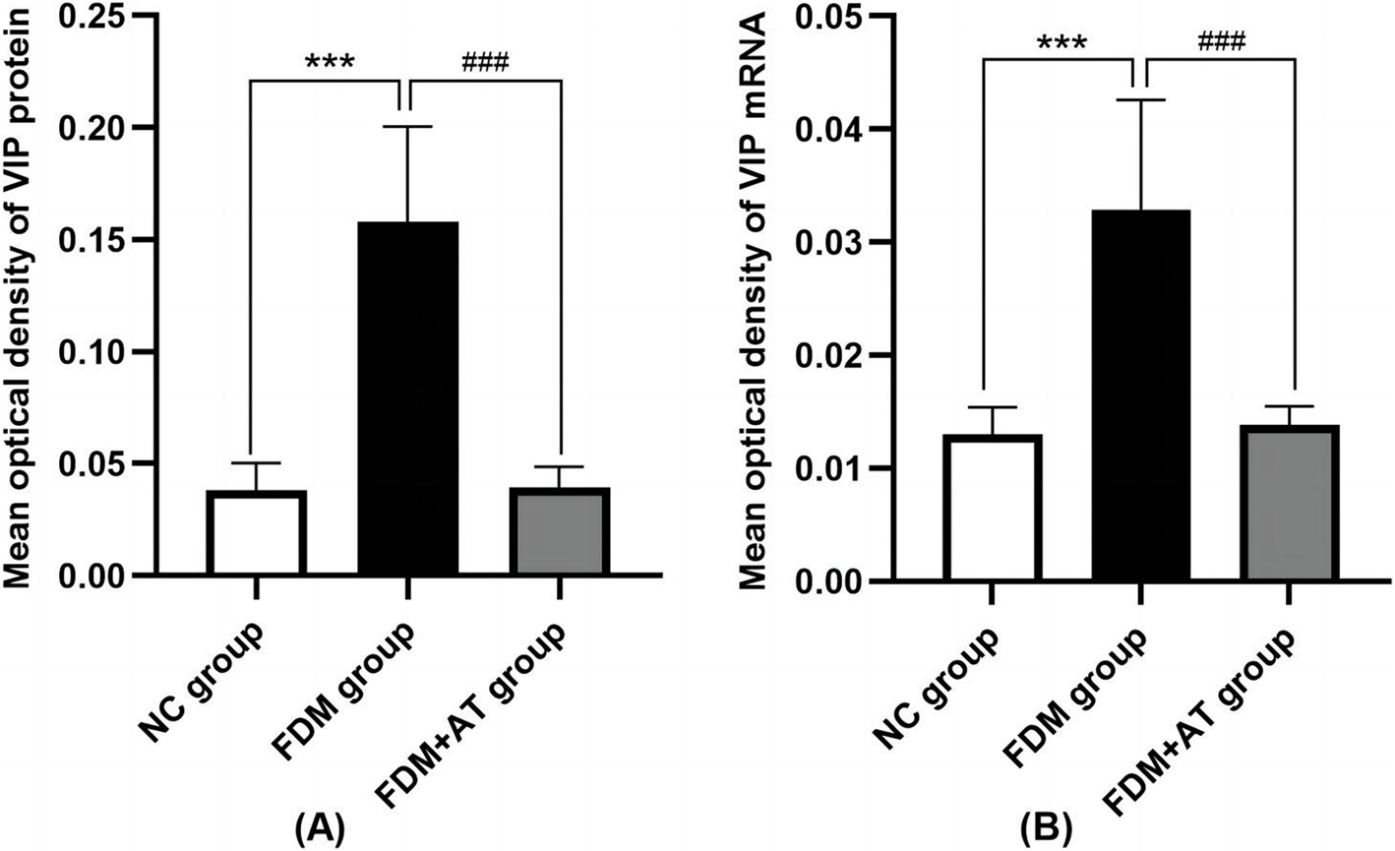
Protein and mRNA expressions of retinal VIP in guinea pig (mean ± SD). Compared with the NC group, the mean optical density of retinal VIP protein and VIP mRNA positive cells in the FDM group were significantly up-regulated (*P* < 0.01). In contrast, those in the FDM + AT group did not change significantly (*P* > 0.05)

Retinal VIP's mRNA and protein expression levels were assessed for four weeks after form deprivation. VIP signals can be observed in the retina of all treatment groups, with expression mainly located in the nerve fiber layer (NFL), ganglion cell layer (GCL), inner plexiform layer (IPL), inner nuclear layer (INL), and photoreceptor outer segment (POS). Compared with the NC group, the FDM group showed a significant increase in both VIP protein and mRNA (*P* < 0.01), while those in the FDM + AT group did not change significantly (*P* > 0.05) (Table 3, Figs. 5, 6 and 7).

**Table 3:** Protein and mRNA expressions of retinal VIP in guinea pig

	NC	FDM	FDM + AT
IHC	0.038271 ± 0.012142^###^	0.157880 ± 0.042568^***^	0.039503 ± 0.009082^###^
ISH	0.013041 ± 0.002360^###^	0.032904 ± 0.009690^***^	0.013807 ± 0.001666^###^

Compared with the NC group,*P < 0.05, **P < 0.01, ***P < 0.001. Compared with the FDM group,^#^*P* < 0.05, ^##^*P* < 0.01, ^###^*P* < 0.001

## Discussion

The present investigation revealed that four weeks of form deprivation resulted in a noticeable shift towards myopia, characterized by a considerable rise in both the degree of myopia and the axial length. Simultaneously, there was a notable increase in the expression of retinal VIP compared to eyes that were considered normal. Nevertheless, the administration of atropine has been found to have a noteworthy impact in mitigating the myopic shift caused by form deprivation. In addition, it has been observed to decrease the growth of myopia progression and axial length elongation while restoring the expression of retinal VIP to normal levels. The findings of this study indicate that augmented expression of VIP may plays a role in the onset and progression of myopia. Furthermore, it is proposed that atropine may regulate myopia by modulating the signaling pathways associated with VIP.

Increasing evidence indicates that local visual processing and signaling mechanisms within the retina regulate the pathogenesis of myopia [25, 26]. Changes in the structure and function of the retina (e.g., neurons, neuronal pathways, and neurotransmitters) are closely related to the occurrence and development of myopia [27, 28]. Transformation of visual stimuli into visual signals in the retina can affect eye growth. Sclera remodeling has been promoted by processing visual signals via multiple signaling pathways or multi-layer signal cascades [29]. In other terms, the retina is essential to the development of vision. Consequently, investigating the interaction between the retinal signaling molecule VIP and the anti-myopia drug atropine promotes the identification of new therapeutic targets and insights for the prevention and treatment of myopia.

The structural and refractive characteristics of the form-deprivation myopia model are very similar to those of spontaneous myopia in humans, and have a relatively small correlation with the central nervous system, which can better reflect retinal-origin local changes [30]. Guinea pigs are ideal animals for inducing form-deprivation myopia, with docile personality, high cooperation, low price, and easy breeding, etc., and can induce high myopic degrees in a short time. Traditional form deprivation is often achieved by suturing the eyelids or using an opaque goggle glued to the skin around the eye. These two methods may lead to eyelid damage, erosion, or infection, which affects the research results. In our study, we used semi-transparent latex balloons to cover the eyes, causing retinal image blurring and inducing myopia. This method avoids mechanical pressure to the anterior segment, significantly reduces animal pain, and is safe, effective, and non-invasive. Yang et al. [24] covered the right eyes of guinea pigs with semi-transparent latex balloons, and after 2 weeks of form-deprivation, there was a significant myopic shift, resulting in -4.41 ± 1.16 D myopia after 4 weeks. Zhong et al. [23] also used this method to develop a myopia of -4.33 ± 0.67D in 3-week-old guinea pigs after 4 weeks of form-deprivation.

VIP is expressed in the retina and the visual cortex and plays an essential role in visual development [31, 32]. Recently, studies based on different animal models of myopia, including monkeys [21], chickens [22, 33], and mice [18, 31], revealed that the VIP signal cascade mediates the occurrence and development of myopia. VIP expression is closely related to regulating eye development, with a positive correlation to vitreous chamber depth specifically shown [21, 33]. The biological effect of VIP in the retina is mainly mediated via binding to VIP receptor 2 (VIP-R2), which has been identified as a candidate gene for susceptibility to high myopia [34, 35]. Amacrine cells in the retina can secrete VIP [36], which has been shown to have potent antioxidant, anti-apoptotic, and anti-inflammatory properties and the ability to promote proliferation and differentiation in retinal cells [31, 37]. VIP can increase the activity of retinal neurons and has potent neurotrophic and protective properties, particularly in cases of retinal injury induced by ischemia, oxidation, and inflammation [38–40]. VIP can activate the activity-dependent neuroprotective protein (ADNP) and its short peptide NAP either directly or indirectly [41]. These substances can prevent apoptosis of retinal cells and promote neuron remediation [42]. VIP may be up-regulated via autonomous negative feedback to provide nutritional support and neuroprotection to the retinal cell population when form deprivation affects the growth and development of the eyes and compromises the proliferation and differentiation of retinal cells. Moreover, VIP, a non-cholinergic vasodilator, can induce relaxation in ocular blood vessels, augment blood circulation in the choroid, and ultimately modulate the retinal blood supply [43]. Xiangtian Zhou's team found significant reductions in ChT and choroidal blood perfusion (ChBP) in FDM guinea pigs, and they both increased during recovery. Changes in ChT were positively correlated with changes in ChBP and decreased ChBP leads to scleral hypoxia [44, 45]. When form deprivation reduces choroidal blood flow and causes retina-choroid-sclera ischemia and hypoxia [44], retinal neurons may secrete VIP to improve ocular fundus ischemia and hypoxia symptoms. As a result, increased retinal VIP expression may constitute a self-protection strategy against retinal injury. H&E staining, on the other hand, demonstrated that up-regulation of VIP expression did not significantly alleviate form deprivation-induced choroid blood flow reduction, and the ChT, choroidal blood vessel lumen and area of form-deprived eyes remained smaller than that of normal eyes. This is inconsistent with our expectations since we observed increased VIP expression correlating with reduced choroidal blood vessel lumen and area in form-deprived eyes and reversal of these trends upon atropine treatment. As a result, we hypothesize that upregulated VIP may contribute to enhancing choroidal blood flow in FDM, but it cannot completely resist choroidal ischemia and hypoxia caused by form deprivation. Alternatively, the VIP pathway alone may not cause vasodilation, and other pathways or components may be involved in regulating choroid blood flow in FDM. However, direct evidence for this is still lacking, and further explorations are required. Intracellularly, VIP signals via cAMP, which is shared with dopamine (DA) signaling in the retina [14, 15], allowing DA to regulate VIP signaling directly. DA activation of Dopamine D2-like receptors inhibits cAMP signaling and down-regulates the expression of retinal VIP [46, 47]. DA can also activate Ca^2+^ channels to antagonize VIP expression [48]. Form deprivation reduces DA secretion in the retina [18]. This likely leads to increased cAMP signaling or activation of the Ca^2+^ channel and subsequent increased VIP in the retina. Increased VIP can affect cell metabolism and neuronal activity [49], accelerate eyeball growth, increase axial length, and eventually contribute to axial myopia.

In this study, we found VIP expression in the retina, and form deprivation up-regulated the mRNA and protein expressions of retinal VIP. This is consistent with several studies demonstrating a relationship between form deprivation and up-regulated VIP expression [18, 21, 50, 51]. However, other FDM models have shown reduced (primate [52]; chicken [22]) or negligible changes (mouse [19, 20]) in the mRNA expression level of retinal VIP. These inconsistencies may be attributed to the experimental animal species, deprivation time, and degree of myopia.

Atropine is a non-selective muscarinic acetylcholine receptor blocker that can relax smooth muscles, relieve spasms, dilate the pupil, and induce cycloplegia [53]. It is currently the most widely used drug for myopia control. The effect of atropine on myopia control is concentration-dependent, the higher the concentration, the better the control effect of refraction and axial length, and high-dose atropine (usually 1%) is the most effective [54–56]. This is the reason why 1% concentration of atropine was chosen first for our study. Although various clinical and animal studies have demonstrated the safety and efficacy of atropine for myopia, the specific mechanism of myopia inhibition remains unclear [57]. It was initially thought to be related to cycloplegia of the ciliary muscle. However, subsequent research in the chick model suggested alternative mechanisms since the ciliary muscle of chickens is not dominated by muscarinic receptors [58]. This study demonstrated that atropine effectively inhibits experimental myopia caused by form deprivation and reduces the observed structural degeneration in the retina, choroid, and sclera. This is consistent with some earlier studies, which also suggested that the retina, choroid, and sclera are possible atropine action sites. Additionally, we found that atropine significantly reduced retinal VIP expression in form-deprived eyes. Czepita et al*.* [59]reported that VIP antagonists can inhibit animal experimental myopia. Therefore, we speculated that atropine may directly or indirectly affect retinal VIP expression, regulate information transmission within the retina-choroid-sclera pathway, and ultimately affect scleral remodeling. Atropine may regulate VIP expression via dopamine or acetylcholine (ACh). Atropine has been shown to stimulate the synthesis and release of retinal DA [60, 61], which interacts with VIP, as discussed earlier. Atropine is a competitive inhibitor of ACh at the muscarinic receptor [62]. ACh can directly stimulate VIP neurons and promote VIP release. Fahrenkrug et al*.* [63] found that systemic injection of ACh induces gastrointestinal release of VIP, which can be blocked by atropine. Atropine may also inhibit myopia development by altering choroidal blood flow. Previous research has demonstrated that the administration of atropine has the potential to induce a notable augmentation in both ChT and ChBP, as evidenced by animal [64]and clinical [65] trials. However, the precise underlying mechanism responsible for this observed effect remains uncertain. The results of our morphological research indicate that the administration of atropine leads to a considerable increase in the ChT, choroidal blood vessel lumen and area. This finding suggests that atropine has the potential to enhance choroidal blood flow. Zhu et al. observed a decrease in choroidal vessel area and ChT in myopic guinea pigs, with these changes being attenuated by atropine [56]**.** It is worth noting that VIP functions as a non-cholinergic vasodilator in addition to its other properties. This characteristic allows VIP to induce relaxation in the eye's blood vessels and enhance the flow of blood in the choroidal region [43]. Thus, the stimulatory effect of atropine on choroidal blood vessels and its inhibitory effect on VIP expression suggests that atropine regulation of the choroid may not require VIP, and more research is required to delineate the exact mechanisms. Beyond the eye, the development of the visual cortex may also be regulated by interaction between atropine and VIP since atropine has been shown to inhibit VIP synthesis in the visual cortex [49]. As a result, atropine could regulate myopia development via several pathways in the eye and visual cortex, with VIP perhaps serving as a central signaling molecule in this process.

Wu and his colleagues [66], as well as Zhong [23], have discovered in experimental models of myopia that the retinal thickness decreases in myopic guinea pigs. Additionally, Kim and others [67], as well as Wu and his team [68], have found through clinical studies that the retinal thickness also decreases in individuals with myopia. Our study indicates that compared to the normal group, the majority of myopic guinea pigs in the FDM group exhibited a decrease in retinal thickness. However, not all guinea pigs in the FDM group experienced this decrease. We speculate that this may be due to the relatively large size of the guinea pig eyes, making it challenging to achieve complete fixation. The detached retinal tissue may be deformed by pulling, affecting accurate measurement of retinal thickness. Therefore, we chose to exclude quantitative evaluation of retinal thickness. Perhaps, the Optical Coherence Tomography Angiography (OCTA) technology can help us dynamically monitor the changes in both retinal and choroidal thickness in living animals, avoiding the effects of histopathological processing and enabling more rigorous and comprehensive experimental conclusions. In future studies, we will continue to explore and refine this method.

Several aspects of this study demand additional improvements. First, it is worth noting that while this study employed an untreated control group, a more suitable internal matched control may have been the unaffected eye of the FDM guinea pigs. Additionally, the exclusive utilization of the atropine group would have allowed for a more comprehensive examination of the effects of the drug on retinal VIP. Second, titrating the dose of atropine will provide more insights into the mechanism of atropine than the fixed, high-dose atropine used in the current experiment. Third, morphological studies of the choroid revealed choroid thickness and area changes. Still, they did not provide a direct readout for lumen diameter, area, and blood flow, which microangiography techniques can more accurately measure. Furthermore, the histopathological processing may have some effects on retinal thickness, morphology, and other parameters. Therefore, the study only conducted a qualitative analysis of retinal morphological modifications, without a thorough quantitative analysis of factors such as retinal cell quantity and retinal thickness. Finally, Both retinoscopy and axial length by ultrasonic biometry are known to be dependent on subjective variation and this itself adds to a majority of confounders in the study.

## Conclusions

This study represents the first exploration of the impact of atropine on retinal VIP expression using the FDM guinea pig model. Our research has contributed supplementary evidence to substantiate the efficacy of atropine as a pharmacological intervention for myopia. Additionally, we have identified the retinal VIP signaling pathway as a crucial target for preventing and managing myopia. Furthermore, our study has shed light on the involvement of VIP in visual development and the regulatory role of atropine in myopia. Future studies should further delineate the mechanism of interactions between atropine and VIP and their impacts on the eye and the visual cortex during myopia development.

## Data Availability

The datasets used and analyzed during the current study are available from the corresponding author upon reasonable request. All relevant datasets related to the study can be found in the specified (https://figshare.com/s/e41466e351595b6055f6).

## Abbreviations

VIP: Vasoactive intestinal polypeptide
FDM: Form-deprivation myopia
DA: Dopamine
cAMP: Cyclic Adenosine monophosphate
VIP-R2: VIP receptor 2
ACh: Acetylcholine
ADNP: Activity-dependent neuroprotective protein
IHC: Immunohistochemistry
ISH: In situ hybridization
H&E: Hematoxylin and eosin
ChT: Choroidal thickness
ChBP: Choroidal blood perfusion
OCTA: Optical coherence tomography angiography
